# Functional Redundancy and Specialization of the Conserved Cold Shock Proteins in *Bacillus subtilis*

**DOI:** 10.3390/microorganisms9071434

**Published:** 2021-07-02

**Authors:** Patrick Faßhauer, Tobias Busche, Jörn Kalinowski, Ulrike Mäder, Anja Poehlein, Rolf Daniel, Jörg Stülke

**Affiliations:** 1 Department of General Microbiology, GZMB, Georg-August-University Göttingen, 37077 Göttingen, Germany; patrick.fasshauer@uni-goettingen.de; 2 Center for Biotechnology (CeBiTec), Bielefeld University, 33615 Bielefeld, Germany; tbusche@cebitec.uni-bielefeld.de (T.B.); joern@cebitec.uni-bielefeld.de (J.K.); 3 Interfaculty Institute for Genetics and Functional Genomics, University Medicine Greifswald, 17487 Greifswald, Germany; ulrike.maeder@uni-greifswald.de; 4 Department of Genomic and Applied Microbiology, GZMB, Georg-August-University Göttingen, 37077 Göttingen, Germany; apoehle3@gwdg.de (A.P.); rdaniel@gwdg.de (R.D.)

**Keywords:** *Bacillus subtilis*, cold shock proteins, quasi-essential

## Abstract

Many bacteria encode so-called cold shock proteins. These proteins are characterized by a conserved protein domain. Often, the bacteria have multiple cold shock proteins that are expressed either constitutively or at low temperatures. In the Gram-positive model bacterium *Bacillus* *subtilis*, two of three cold shock proteins, CspB and CspD, belong to the most abundant proteins suggesting a very important function. To get insights into the role of these highly abundant proteins, we analyzed the phenotypes of single and double mutants, tested the expression of the *csp* genes and the impact of CspB and CspD on global gene expression in *B. subtilis*. We demonstrate that the simultaneous loss of both CspB and CspD results in a severe growth defect, in the loss of genetic competence, and the appearance of suppressor mutations. Overexpression of the third cold shock protein CspC could compensate for the loss of CspB and CspD. The transcriptome analysis revealed that the lack of CspB and CspD affects the expression of about 20% of all genes. In several cases, the lack of the cold shock proteins results in an increased read-through at transcription terminators suggesting that CspB and CspD might be involved in the control of transcription termination.

## 1. Introduction

*Bacillus subtilis* is the model organism for a large group of Gram-positive bacteria, among them serious pathogens and biotechnologically relevant bacteria. Due to this relevance, *B. subtilis* is one of the best-studied organisms. However, for about 25% of all proteins, the function is completely unknown, or the proteins are only poorly characterized [1]. Similarly, in the artificially created genome of the minimal organism *Mycoplasma mycoides* JCVI-syn3.0, about one-third of all encoded proteins are of unknown function [2]. These numbers demonstrate how far we are from a complete understanding of even seemingly simple model organisms.

We are interested in a comprehensive understanding of *B. subtilis*. Obviously, the large fraction of unknown proteins is a major challenge. Among the unknown and poorly characterized proteins, the large majority is not or only weakly expressed under standard growth conditions and probably only important under very specific conditions [3]. However, among the 100 most abundant proteins, there are six proteins for which no clear function has been identified [4]. These highly abundant unknown proteins are likely to be of major relevance for the cell, and their functional analysis should be given a high priority. Among these proteins are the cold shock proteins CspB and CspD, which are thought to act as RNA chaperones in *B. subtilis* [5]. The cold shock proteins are a large protein family that are all characterized by a conserved cold shock domain (see [6] for a phylogenetic tree). Among the bacteria, the cold shock proteins are nearly ubiquitous, and most bacteria encode multiple cold shock proteins. *B. subtilis* has three cold shock proteins, CspB, CspC, and CspD. The genes encoding the *B. subtilis* cold shock proteins are highly expressed under a wide variety of growth conditions, and with 31,200 and 21,500 protein molecules per cell, CspB and CspD, respectively, even belong to the 15 most abundant proteins in *B. subtilis* [4,7].

The presence of multiple closely related proteins in an organism always raises the question of whether they participate in the same function or if their specific activity is slightly different. There are a few examples of families of closely related proteins in *B. subtilis*. In the case of ABC transporters or classic amino acid and ion transporters, both functional overlaps, as well as specific functions, have been observed [8,9,10]. In some cases, as described for the three diadenylate cyclases, all proteins have the same enzymatic activity, but their expression and activities are differentially controlled [11,12]. Similarly, the four DEAD-box RNA helicases have distinct domains in addition to the active protein core, and these proteins have independent functions [13]. Finally, in some cases, regulatory systems consisting of multiple parts, such as two-component regulatory systems, ECF sigma factors, PTS-controlled RNA-binding antitermination proteins, or stress signaling proteins, specificity is achieved by co-evolving interacting partners of these systems [14,15,16,17].

For the *B. subtilis* cold shock proteins, it has been observed that the bacteria are not viable in the absence of all three proteins [5]. This, on the one hand, supports the idea that these highly abundant proteins play a major role in the *B. subtilis* cell and, on the other hand, suggests that there is at least some functional overlap between them. In *Staphylococcus aureus*, the CspA protein is unique among the three cold shock proteins since it is the only one that is strongly constitutively expressed and important for the expression of the virulence factor staphyloxanthin [18,19]. It was shown that a single amino acid in CspA, a conserved proline residue, is responsible for this regulatory effect [18].

To get more insights into the function(s) of the cold shock proteins of *B. subtilis*, we studied their expression as well as phenotypes of mutants. The deletion of *cspB* and *cspD* was found to result in the rapid acquisition of suppressor mutations that often result in increased expression of *cspC*, suggesting that CspC might at least partially take over the function of CspB and CspD if it is overexpressed. We also studied the global RNA profile of a strain lacking CspB and CspD and observed that these proteins are important for transcription elongation and termination and that they can control the read-through at transcription terminators.

## 2. Materials and Methods

### 2.1. Bacterial Strains, Growth Conditions, and Phenotypic Characterization

All *B. subtilis* strains used in this study are listed in Table 1. All strains are derived from the laboratory strain 168 (*trpC2*). *B. subtilis* was grown in Lysogeny Broth (LB medium) [20]. LB plates were prepared by addition of 17 g Bacto agar/l (Difco) [20,21]. Quantitative studies of *lacZ* expression in *B. subtilis* were performed as described previously [20]. One unit of β-galactosidase is defined as the amount of enzyme which produces 1 nmol of o-nitrophenol per min at 28 °C.

### 2.2. DNA Manipulation and Genome Sequencing

*B. subtilis* was transformed with plasmids, genomic DNA, or PCR products according to the two-step protocol [20,21]. Transformants were selected on LB plates containing erythromycin (2 µg/mL) plus lincomycin (25 µg/mL), chloramphenicol (5 µg/mL), kanamycin (10 µg/mL), tetracycline (12.5 µg/mL), or spectinomycin (250 µg/mL). S7 Fusion DNA polymerase (Biozym, Hessisch Oldendorf, Germany) was used as recommended by the manufacturer. DNA fragments were purified using the QIAquick PCR purification kit (Qiagen, Hilden, Germany). DNA sequences were determined by the dideoxy chain termination method [21]. Chromosomal DNA from *B. subtilis* was isolated using the peqGOLD Bacterial DNA Kit (Peqlab, Erlangen, Germany). To identify the mutations in the suppressor mutant strains GP1989, GP1990, and GP2900, the genomic DNA was subjected to whole-genome sequencing as described previously [3]. The reads were mapped on the reference genome of *B. subtilis* 168 (GenBank accession number: NC_000964) [23]. Mapping of the reads was performed using the Geneious software package (Biomatters Ltd., Auckland, New Zealand) [24]. The resulting genome sequences were compared to that of our in-house wild-type strain. Single nucleotide polymorphisms were considered as significant when the total coverage depth exceeded 25 reads with a variant frequency of ≥90%. All identified mutations were verified by PCR amplification and Sanger sequencing.

### 2.3. Construction of Translational LacZ Reporter Gene Fusions

Plasmid pAC5 [25] was used to construct translational fusions of the *cspB*, *cspC*, and *veg* promoter regions with the *lacZ* gene. For this purpose, the respective promoter regions were amplified using the oligonucleotide pairs PF151/PF152 (*cspB*, corresponds to 379 bp upstream and 5 bp downstream of the ATG start codon, respectively), PF97/PF98 (*cspC*, 237 bp upstream, 125 bp downstream), and PF127/PF118 (*veg*, 280 bp upstream, 50 bp downstream), and chromosomal DNA of *B. subtilis* 168 as the template. The PCR products were digested with EcoRI and BamHI and cloned into pAC5 linearized with the same enzymes. The resulting plasmids were pGP3136, pGP3117, and pGP3133 for the *cspB*, *cspC*, and *veg* promoters, respectively. Plasmids pGP3119 and pGP3134 for the *cspC* and *veg* mutant upstream regions were constructed accordingly using chromosomal DNA of the suppressor mutants GP1986 and GP1990 as the template, respectively. The promoter region of *cspD* (194 bp upstream, 30 bp downstream) was fused to *lacZ* using a PCR product constructed with oligonucleotides PF246/PF247 and PF250/PF251 using pAC5 as a template and PF248/PF249 with chromosomal DNA from *B. subtilis* 168 as a template. The fragments were joined and amplified in a PCR as described previously for the deletion of genes [26,27]. The fusion constructs were integrated into the *amyE* site of the *B. subtilis* chromosome by the transformation of *B. subtilis* 168 with linearized plasmids or the PCR product. The resulting strains were GP3283 (*cspB* wild type promoter), GP1984 (*cspC* wild type promoter), GP1986 (*cspC* mutant promoter), GP3286 (*cspD* wild type promoter), GP2898 (*veg* wild type promoter), and GP2899 (*veg* mutant promoter).

### 2.4. Construction of Mutants

Deletion of the *csp* genes was achieved by transformation with PCR products constructed using oligonucleotides (see Table S1) to amplify DNA fragments flanking the target genes and intervening antibiotic resistance cassettes as described previously [26,27]. The strain GP3274 harboring the CspC-A58P variant was created using the multiple-mutation reaction method, as described previously [28], to generate the *cspC* fragment followed by the combination of the fragment with the downstream flanking region and a spectinomycin resistance cassette as described above.

### 2.5. RNA-Sequencing

*B. subtilis* 168 wild type and the *cspB cspD* double mutant GP1971 were grown in LB medium to an OD_600_ of 0.2 and were harvested by mixing them 5:3 with frozen killing buffer (20 mM Tris-HCl pH 7.5, 6 mM MgCl_2_, 20 mM NaN_3_) followed by snap-freezing of the pellets in liquid nitrogen. RNA isolation and quality assessment were performed as described previously [7] with an additional DNase I treatment using TURBO DNase (Ambion). The RNA quality was checked by Trinean Xpose (Trinean, Gentbrugge, Belgium) and the Agilent RNA Nano 6000 kit using an Agilent 2100 Bioanalyzer (Agilent Technologies, Böblingen, Germany). The Ribo-Zero rRNA Removal Kit (Bacteria) from Illumina (San Diego, CA, USA) was used to remove the rRNA. The TruSeq Stranded mRNA Library Prep Kit from Illumina was applied to prepare the cDNA libraries. Final libraries were sequenced paired-end on an Illumina MiSeq system (San Diego, CA, USA) using 75 bp read length.

Trimmed reads were mapped to the *B. subtilis* 168 genome sequences (NCBI GenBank accession number AL009126.3) using Bowtie2 [29]. The annotation AL009126.3 from the NCBI RefSeq database was augmented with the RNA features previously annotated [7]). In order to perform differential gene expression analysis, DEseq2 [30] was used as a part of the software ReadXplorer v2.2 [31]. Statistically significant expression changes (adjusted *p*-value ≤ 0.01) with log2 fold change >1.0 or <−1.0 were used. RNA-seq data have been deposited in the ArrayExpress database at EMBL-EBI (www.ebi.ac.uk/arrayexpress (accessed on 2 July 2021)) under accession number E-MTAB-10658.

### 2.6. Qualitative PCR and Real-Time Quantitative Reverse Transcription PCR

For RNA isolation, the cells were grown in LB medium and harvested at an OD_600_ of 1.2 for qualitative PCR and an OD_600_ of 0.5–0.8 for quantitative RT-PCR. Preparation of total RNA was carried out as described previously [32]. cDNAs for qualitative PCR were synthesized using the RevertAid First Strand cDNA Synthesis Kit from ThermoFisher according to the manufacturer’s instructions. cDNAs for qRT-PCR were synthesized using the One-Step RT-PCR kit (BioRad, Feldkirchen, Germany) as described [33]. qRT-PCR was carried out on the iCycler instrument (BioRad), following the manufacturer’s recommended protocol by using the primers indicated in Table S1. The *rpsE* and *rpsJ* genes encoding constitutively expressed ribosomal proteins were used as internal controls. Data analysis and the calculation of expression ratios as fold changes were performed as described [33]. qRT-PCR experiments were performed in triplicate.

### 2.7. Microscopy

For microscopy, cells were grown at 37 °C in liquid LB medium overnight. The overnight culture was directly used for microscopy or for inoculation of LB medium, which was incubated at 37 °C to an OD_600_ of 0.3–0.5. Images were acquired using an Axioskop 40 FL fluorescence microscope, equipped with digital camera AxioCam MRm and AxioVision Rel (version 4.8, Carl Zeiss, Oberkochen, Germany) software for image processing (Carl Zeiss, Göttingen, Germany) and Neofluar series objective at ×100 primary magnification.

## 3. Results

### 3.1. Relative Contribution of the Cold Shock Proteins to the Growth of B. subtilis

**Figure 1:**
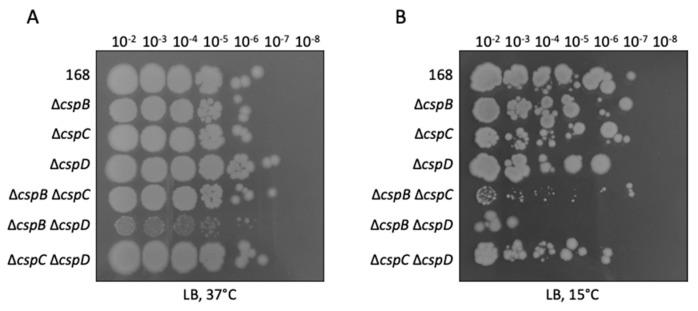
Growth of *csp* knockout mutants. The wild-type strain *B. subtilis* 168 and the *csp* mutants GP1968 (∆*cspB*), GP1969 (∆*cspC*), GP2614 (∆*cspD*), GP1970 (∆*cspB* ∆*cspC*), GP1971 (∆*cspB* ∆*cspD*), and GP1972 (∆*cspC* ∆*cspD*) were cultivated on LB-agar (**A**) at 37 °C for one day and (**B**) at 15 °C for 11 days. The results are representative of three biological replicates.

It has been reported that *B. subtilis* is not viable in the absence of the three cold shock proteins CspB, CspC, and CspD [5]. To get better insights into the role(s) of the individual cold shock proteins, we constructed a set of single and double mutants. In agreement with the published data, deletion of all three *csp* genes was not possible. First, we compared the growth of the strains on plates at 37 °C and 15 °C. As shown, in Figure 1A, at 37 °C, all single mutants, as well as the *cspB cspC* and *cspC cspD* double mutants, grew indistinguishable from the wild-type strain *B. subtilis* 168. In contrast, the *cspB cspD* double mutant GP1971 barely formed colonies; instead, this mutant acquired suppressor mutations that restored growth (Figure 1A, see below). At 15 °C, growth of the *cspB cspC* double mutant was also strongly impaired comparable to the *cspB cspD* double mutant (Figure 1B), whereas the *cspC cspD* double mutant showed a slight growth defect.

**Figure 2:**
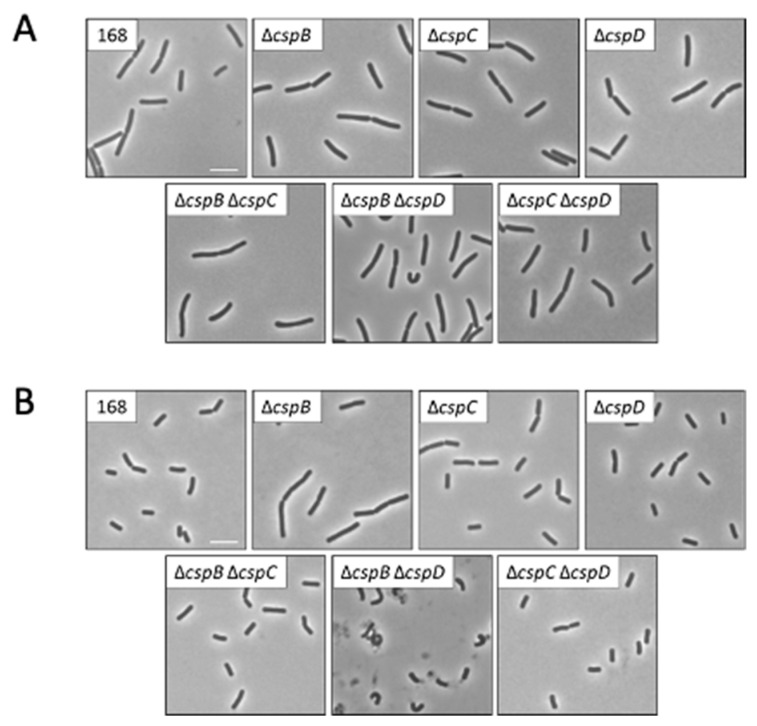
Phenotypic analysis of *B. subtilis csp* mutants. The wild-type strain *B. subtilis* 168 and the csp mutants GP1968 (∆*cspB*), GP1969 (∆*cspC*), GP2614 (∆*cspD*), GP1970 (∆*cspB* ∆*cspC*), GP1971 (∆*cspB* ∆*cspD*), and GP1972 (∆*cspC* ∆*cspD*) were analyzed for morphology by phase-contrast microscopy. Scale bars, 5 µM. (**A**) Cells were cultivated in liquid LB medium at 37 °C to an OD_600_ of 0.3–0.5. (**B**) Cells were cultivated in liquid LB medium at 37 °C overnight to the stationary growth phase.

Microscopic analysis of the cell morphologies of the different csp mutant strains indicated that the cold shock proteins did not affect cell morphology during logarithmic growth (see Figure 2A). Similarly, loss of *cspC* or *cspD* did not affect the morphology in the stationary phase. In contrast, the deletion of *cspB* in GP1968 resulted in the formation of elongated cells in the stationary phase suggesting a defect in cell wall biosynthesis and/or cell division or in the entry to stationary phase (Figure 2B). The *cspB cspD* double mutant showed a highly aberrant cell morphology with curly cells in the stationary phase (see Figure 2B). The strong phenotype of the *cspB cspD* double mutant is in good agreement with the poor growth of this mutant.

In our attempts to combine the different mutations by genetic transformation, we observed that the *cspB cspD* double mutant GP1971 had completely lost genetic competence, whereas all single mutants, as well as the other double mutants, were not affected.

Taken together, these data indicate that none of the individual cold shock proteins is essential for the viability of *B. subtilis*. Moreover, strains expressing either CspB or CspD exhibited few phenotypic effects. In contrast, the simultaneous loss of the latter two proteins had drastic consequences for growth, cell morphology, and genetic competence. This suggests that the two proteins have similar and overlapping activities and that their function cannot be taken over by CspC.

In several cases, such as for diadenylate cyclases and undecaprenyl pyrophosphate phosphatases of *B. subtilis*, the proteins have very similar functions but are unable to replace each other because they are expressed under different conditions [12,34,35]. In contrast, all cold shock genes are highly expressed under all conditions [1,7], and CspB and CspD do even belong to the most abundant proteins in *B. subtilis* [4]. To get more insights into the expression of the three *csp* genes in *B. subtilis*, we constructed and analyzed fusions of their promoter regions to a promoterless *lacZ* reporter gene encoding β-galactosidase. As shown in Table 2, all three genes were highly expressed at both 15 °C and 37 °C. However, the expression of the *cspB* and *cspD* genes did not respond to the temperature, whereas *cspC* expression was about five times higher at 15 °C as compared to the expression at 37 °C. Thus, based on the expression response to temperature, only *cspC* can be called a cold shock protein. The high constitutive expression of *cspB* and *cspD* is in excellent agreement with the central function of the encoded proteins in *B. subtilis*.

### 3.2. Suppressor Analysis of the CspB CspD Double Mutant

As mentioned above, the *cspB cspD* double mutant GP1971 was impaired in growth and formed suppressor mutants after two days of incubation on plates. In order to get more insights into the function of these cold shock proteins, we isolated and characterized a set of suppressor mutants that was able to grow in the absence of CspB and CspD. Three of these mutants were subjected to whole-genome sequencing. Each of the strains carried a single point mutation. In the case of strain GP1989, we identified a point mutation upstream of the *cspC* gene. Strain GP1990 had a mutation upstream of the constitutively expressed *veg* gene, and GP2900 carried a mutation affecting the sensor kinase DegS that resulted in a substitution of Pro-245 by serine. We then analyzed three additional suppressor mutants for mutations in the *cspC* and *veg* upstream regions as well as in the *degS* coding sequence. They all carried mutations upstream of *cspC*, highlighting the importance of this mutation, whereas no mutations potentially affecting *veg* and *degS* were found. Since the *csp* genes are not part of the regulon controlled by the DegS-DegU two-component system, we focused our further analyses on the mutations in the upstream regions of *cspC* and *veg*.

**Figure 3:**
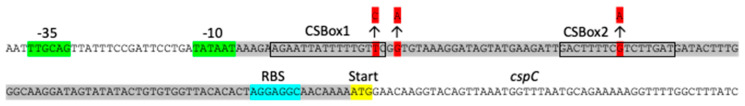
Genetic organization of the *cspC* promoter region. Sequence of the promoter region showing the −35 and −10 regions. The 5’UTR (RNA feature S179) is highlighted in grey. CSBoxes, cold shock boxes; RBS, ribosomal binding site; mutations found in ∆*cspB* ∆*cspD* suppressor mutants are highlighted in red.

In four suppressor mutants, we found single point mutations in the 5’ untranslated region of the *cspC* gene (RNA feature S179 [7]). The 5’ UTRs of *cspB* and *cspC* contained two conserved regions that were dubbed cold shock boxes [5]. The identified mutations in the *cspC* upstream regions were located in one of the cold shock boxes or very close to it (see Figure 3). We assumed that these mutations might affect the expression of *cspC*. To test this idea, we fused the G-65A control region of the mutant strain GP1989 to the *lacZ* reporter gene lacking its own transcription and translation signals and compared the expression of the *lacZ* gene to that driven by the wild type control region. For this purpose, the strains GP1984 and GP1986 carrying the *lacZ* gene under the control of the wild type and mutant *cspC* upstream regions, respectively, were cultivated in LB medium at 37 °C, and the resulting β-galactosidase activities were determined. For the wild type, we observed 4360 (±210) units of β-galactosidase activity per mg of protein, whereas the activity was increased to 9450 (±710) units for the mutant *cspC* upstream region. This indicates that the mutation resulted in higher *cspC* expression, which in turn is likely to be the reason for the suppression of the *cspB cspD* double mutant.

**Figure 4:**
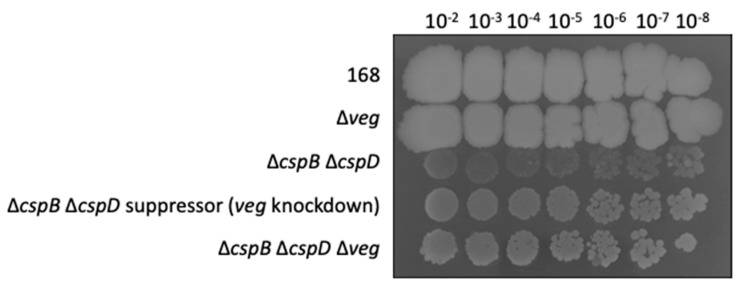
Growth of *veg* knockdown and knockout mutants in the Δ*cspB* Δ*cspD* background. The wild-type strain *B. subtilis* 168, GP2888 (∆*veg*), GP1971 (∆*cspB* ∆*cspD*), GP1990 (∆*cspB* ∆*cspD* suppressor with knockdown mutation of *veg*), and GP2897 (∆*cspB* ∆*cspD* ∆*veg*) were cultivated on LB-agar at 37 °C for 36 h. The results are representative of three biological replicates.

The mutation in the 5’UTR of *veg* affected the ribosomal binding site (GGUGGA → UGUGGA), suggesting reduced expression of the *veg* gene in the suppressor mutant GP1990. Again, we fused the wild type and mutant control regions of *veg* to the *lacZ* reporter gene and compared the β-galactosidase activities. For *B. subtilis* GP2898 (wild type), we observed 34 (±1) units of β-galactosidase activity per mg of protein. In contrast, the activity was reduced to 2 (±1) units of β-galactosidase for the *lacZ* gene under the control of the mutant *veg* 5’ UTR in GP2899. This indicates that a reduced expression of the *veg* gene might help to overcome the deleterious effect of the simultaneous loss of the two major cold shock proteins. To test this idea, we combined the *veg* gene deletion with the *cspB cspD* deletion and observed the growth of the strains on complex medium. While the *cspB cspD* double mutant GP1971 grew only poorly, growth was restored both if the expression of the *veg* gene was reduced or if the *veg* gene was deleted (Figure 4). These observations indicate that the Veg protein may play a role in RNA metabolism in *B. subtilis*, and that it becomes toxic in the absence of the major cold shock proteins.

### 3.3. A Modified CspC Protein Can Take over the Functions of CspB and CspD

**Figure 5:**
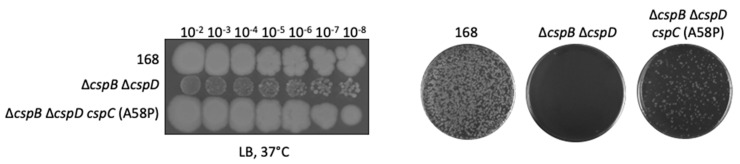
The *cspC* (A58P) variant restores growth and genetic competence in the Δ*cspB* Δ*cspD* background. The left panel shows the strains *B. subtilis* wild type 168, GP1971 (∆*cspB* ∆*cspD*), and GP3275 (Δ*cspB* Δ*cspD cspC* (A58P) that were cultivated on LB-agar at 37 °C for 36 h. The right panel shows the transformants obtained after transformation an equal number of competent cells with 200 ng of chrom. DNA. Data are representative of three biological replicates.

As mentioned above, the three cold shock proteins of *B. subtilis* are highly similar to each other. A recent study on the cold shock proteins of *S. aureus* identified the proline residue at position 58 in CspA as functionally important [18]. This residue is located on the protein’s surface close to the RNA-binding site [18]. The corresponding residue conserved in *B. subtilis* CspB and CspD, but not in CspC. We, therefore, decided to replace the alanine present at this position in CspC with a proline residue and to assay whether the modified protein could compensate for the lack of CspB and CspD. For this purpose, we combined the *cspC* (A58P) allele with the deletions of *cspB* and *cspD*. The resulting strain was GP3275. We then compared GP3275 and the corresponding *cspB cspD* double mutant GP1971 with respect to growth and genetic competence (see Figure 5). As shown, CspC-A58P could compensate for the loss of both CspB and CspD for growth, and partial compensation was observed for genetic competence. This indicates that native expression of the CspC-A58P variant is sufficient for the suppression of the *cspB cspD* double mutant, similar to increased expression of the wild type CspC protein.

### 3.4. RNA-Seq Analysis Suggests a Role for Cold Shock Proteins in Transcription Termination and Elongation

While it is clear from the data presented above that the cold shock proteins play a pivotal role in the physiology of *B. subtilis*, even at 37 °C, nothing is known about the specific functions of these proteins. A study on the *E. coli* cold shock proteins suggested that they act as transcriptional antiterminators [36]. To test whether the cold shock proteins of *B. subtilis* are involved in transcription as well, we performed an RNA-seq analysis with the wild-type strain and the *cspB cspD* double mutant GP1971. This analysis allowed us to not only get insights into the regulation of the *B. subtilis* genes by the cold shock proteins but also to study their impact on the expression of intergenic regions. In total, 542 and 305 genes exhibited an increased and decreased expression in the *cspB cspD* double mutant with more than twofold changes of expression. The most strongly affected transcription units (more than 20-fold change in expression) are listed in Table 3 (see Table S2 for the complete dataset). Among the most strongly upregulated genes were many involved in the utilization of different carbon sources. The most strongly downregulated genes were dominated by genes involved in aerobic and anaerobic regulation. The genes belong to the Rex and Fnr regulons (see Table 3).

**Figure 6:**
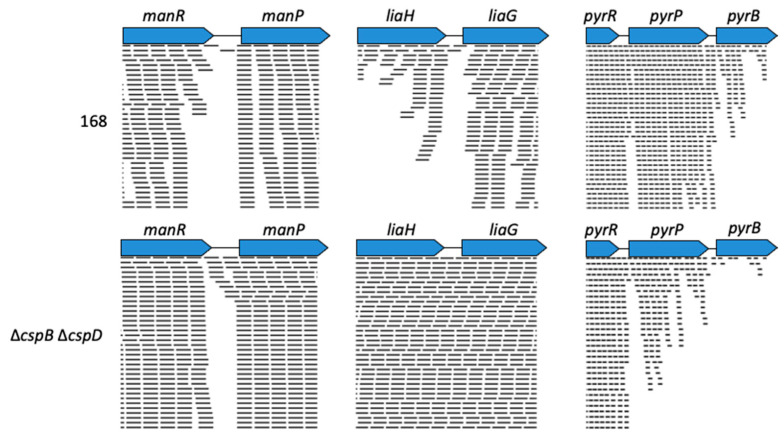
Differences in transcription in 168 and the ∆*cspB* ∆*cspD* mutant. The intergenic regions between *manR-manP*, *liaH-liaG*, *pyrR-pyrP-pyrB* in *B. subtilis* wild type 168 (upper panel) and the ∆*cspB* ∆*cspD* mutant GP1971 (lower panel) aligned with the paired reads generated by RNA-sequencing are shown. The data are representative of three biological replicates. Pictures of aligned reads were created using the Geneious Software version 2020.0.4 (Biomatters Ltd., New Zealand).

As the *E. coli* Csp proteins act as transcriptional antiterminators, we also studied the expression of intergenic regions of the most strongly CspB/CspD responsive genes. Strikingly, in several cases, we observed an increased read-through at transcriptional terminators in the double mutant, e.g., between the *manR* and *manP* genes and the *liaH* and *liaG* genes (see Figure 6). This finding suggests that CspB and CspD support termination instead of acting as antiterminators in these intergenic regions. In contrast, the lack of CspB and CspD resulted in reduced read-through at the transcription termination structures between the *pyrR* and *pyrP* and *pyrP* and *pyrB* genes. The regulation of transcription between these genes involves the binding of the PyrR protein to the intergenic regions in the presence of UMP or UTP. PyrR binding results in the termination of transcription between *pyrR* and *pyrP* and *pyrP* and *pyrB* [37]. The reduced read-through in the absence of CspB and CspD allows two conclusions: the cold shock protein may either act as antiterminator proteins at the *pyr* operon, or they may interfere with the binding of PyrR to its target regions thus allowing a basal read-through in this operon.

### 3.5. Analysis of Csp-Dependent Transcription Read-Through

**Figure 7:**
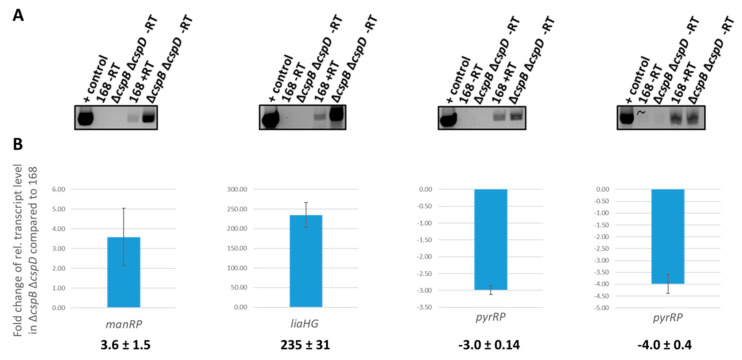
Transcriptional read-through in 168 and the ∆*cspB* ∆*cspD* mutant. (**A**) Qualitative PCR on read-through transcripts. Total RNA was extracted from 168 and GP1971. cDNA was synthesized and served as a template in a PCR with primers annealing up- and downstream of the terminators between *manR-manP*, *liaH-liaG*, *pyrR-pyrP-pyrB*. ‘+ control’: standard PCR using chromosomal DNA of *B. subtilis* 168 as the template, ‘-RT’: control sample without reverse transcriptase, ‘+RT’: sample with reverse transcriptase. (**B**) Fold changes in the expression of read-through transcripts in a ∆*cspB* ∆*cspD* (GP1971) mutant relative to levels in the wild-type strain (168) are shown. RNA was purified from each strain, and quantitative RT-PCR was performed using primer sets amplifying up- and downstream of the terminators in the indicated intergenic regions. Shown values represent the mean of three biological replicates. Errors bars indicate the standard deviations.

To verify the effect of CspB and CspD on transcription read-through, we performed PCR analyses for the intergenic regions of the *man*, the *lia*, and the *pyr* operons. For this purpose, total mRNA was converted to cDNA and used as a template for PCR assays. To make sure that the analysis was specific for the intergenic regions, we amplified regions covering 400 nucleotides upstream and downstream of the transcription terminators. As positive and negative controls, we used assays with chromosomal DNA as the template and assays with mRNA that had not been subjected to reverse transcription, respectively. As shown in Figure 7A, clear products corresponding to the *manR-manR*, *liaH-liaG*, *pyrR-pyrP*, and *pyrP-pyrB* intergenic regions could be observed if the PCR analysis was performed using the cDNA as a template. In the case of the *man* and *lia* operons, the intensity of these products was strongly increased for the *cspB cspD* double mutant GP1971 as compared to the wild-type strain 168. In contrast, little effect of the inactivation of the *cspB* and *cspD* genes was detected for the intergenic regions of the *pyr* operon.

In order to get further evidence for the effect of CspB and CspD on read-through, we performed quantitative RT-PCR analyses for the intergenic regions (see Figure 7B). In agreement with the RNA-seq data and the qualitative PCR analysis, we observed increased read-through in the *cspB cspD* double mutant for the *manR-manP* and *liaH-liaG* intergenic regions. For the intergenic regions of the *pyr* operon, we found reduced read-through, again in agreement with the RNA-seq data.

Thus, our results suggest that CspB and CspD are involved in the control of transcription elongation at terminator structures in B. *subtilis*; however, the increased expression level of the gene upstream of the terminator may also play a role in reduced termination in the *cspB cspD* double mutant.

## 4. Discussion

Cold shock proteins are widespread among bacteria, and many species encode several of these proteins. This is exemplified by the actinobacterium *Lentzea guizhouensis* DHSC013 with as many as 15 distinct *csp* genes [38]. On the other side, some bacteria do not express any cold shock proteins. In the Bacilli, the proteins are ubiquitous, indicating that they are very important for the biology of these bacteria. Our results indicate that the cold shock proteins CspB and CspD performed a quasi-essential function in the cell and suggest that at least one of these proteins should be present in a minimal genome based on *B. subtilis* [39,40].

*B. subtilis* encodes the three cold shock proteins CspB, CspC, and CspD. CspB and CspD are highly similar to each other (81% identical residues). CspC is also similar to both proteins, but the identical residues account only for 71% (with CspD) and 69% (with CspB). The evaluation of the expression as well as the analysis of mutants demonstrated that CspB and CspD are the key cold shock proteins. Actually, the term cold shock proteins is misleading since they are strongly constitutively expressed under a wide range of conditions [7]. Strains lacking one of the cold shock proteins had no or only slightly changed phenotype alterations as compared to the wild type. However, the concomitant loss of the highly similar and constitutively expressed proteins CspB and CspD had severe consequences: growth was strongly impaired, the cells had an aberrant morphology, and lost genetic competence. These observations suggest that CspB and CspD are functionally redundant: at least one of them is required, and a double mutant rapidly acquires suppressor mutations indicating that CspB and CspD together are quasi-essential for *B. subtilis*. In contrast, CspC seemed to play a major role at low temperatures: this was the only cold shock gene that was induced at low temperatures (see Table 2), and double mutants lacking CspC exhibited impaired growth at 15 °C. In the case of the *cspB cspC* double mutant, the growth defect was as severe as observed for the *cspB cspD* mutant, suggesting that CspB is more important than CspD.

Even though CspC seems to be specialized distinctly as compared to CspB and CspD, we observed suppression of the *cspB cspD* double mutant by overexpression of CspC, indicating that CspC can take over the function of the other proteins if it is present at high amounts. Similarly, we have observed suppression of a *topA* mutant lacking the quasi-essential DNA topoisomerase I by overexpression of topoisomerase IV even though the two enzymes are specialized in distinct functions [41]. Interestingly, one amino acid exchange in CspC was sufficient to allow suppression of the *cspB cspD* mutant even at a wild type expression rate: This mutation affects a conserved proline residue that was not present in CspC. Upon introduction of a proline in position 58 of CspC, the protein can functionally replace CspB and CspD, even with respect to genetic competence.

The suppressor analysis also identified the inactivation of the *veg* gene as sufficient to allow the growth of a strain lacking CspB and CspD. The precise function of the constitutively expressed Veg protein has not been identified, but a *veg* mutant was impaired in the expression of biofilm genes and biofilm formation [42]. Interestingly, the Veg protein was recently identified as an mRNA-associated protein in *Clostridium difficile* [22]. Suppression of the *cspB cspD* double mutant by inactivation of Veg suggests that the proteins might act as antagonists.

Two lines of evidence suggest a function related to transcription for CspB and CspD: First, the corresponding *E. coli* proteins play a role in transcription initiation and antitermination [36,43]. Second, a *B. subtilis* strain lacking the quasi-essential endoribonuclease RNase Y readily acquires mutations that affect the RNA polymerase and different transcription factors, such as the transcription elongation factor GreA and the RNA polymerase recycling factor RpoE. In addition to these mutations, inactivation of CspD was found to compensate for the lack of RNase Y [44]. Since all other suppressor mutants affected transcription in *B. subtilis*, it is likely that this was also the case for CspD. The analysis of the transcriptome of the *B. subtilis* *cspB cspD* double mutant confirmed the involvement of these proteins in the expression of about 20% of all genes. The high resolution of the RNA-seq analysis allowed us to interrogate whether the cold shock proteins might have a function at transcription terminators. Indeed, we observed altered read-through at several transcription terminators in the *cspB cspD* mutant. In contrast to the findings for *E. coli*, both our RNA-seq data as well as the PCR-based validation indicated that CspB and CspD favored transcription termination rather than antitermination.

Further studies will have to address the molecular interactions of the cold shock proteins and the specific role of CspC at optimal and low temperatures.

## Figures and Tables

**Table 1:** *B. subtilis* strains used in this study.

Strain	Genotype	Source or Reference
168	*trpC2*	Laboratory collection
GP1968	*trpC2* Δ*cspB*::*cat*	This study
GP1969	*trpC2* Δ*cspC::spec*	This study
GP1970	*trpC2* Δ*cspB::cat* Δ*cspC::spec*	This study
GP1971	*trpC2* Δ*cspB::cat* Δ*cspD::aphA3*	This study
GP1972	*trpC2* Δ*cspC::spec* Δ*cspD::aphA3*	This study
GP1984	*trpC2 amyE::*(P*_cspC_-lacZ cat*)	pGP3117→168
GP1986	*trpC2 amyE::*(P*_cspC_*[G-65A]-*lacZ cat*)	pGP3119→168
GP1989 ^1^	*trpC2* Δ*cspB::cat* Δ*cspD::aphA3* P*_cspC_*-[G-65A]	This study
GP1990 ^1^	*trpC2* Δ*cspB::cat* Δ*cspD::aphA3* P*_veg_*-[G-10T]	This study
GP2614	*trpC2* Δ*cspD::aphA3*	[22]
GP2888	*trpC2* Δ*veg::ermC*	This study
GP2896	*trpC2* Δ*veg::ermC* Δ*cspD::aphA3*	GP2888→GP2614
GP2897	*trpC2* Δ*veg::ermC* Δ*cspB::cat* Δ*cspD::aphA3*	GP1968→GP2896
GP2898	*trpC2 amyE::*(P*_veg_*-*lacZ cat*)	pGP3133→168
GP2899	*trpC2 amyE::*(P*_veg_*-[G-10T]-*lacZ cat*)	pGP3134→168
GP2900 ^1^	*trpC2* Δ*cspB::cat* Δ*cspD::aphA3 degS*[P245S]	This study
GP3251	*trpC2* Δ*cspB::tet*	This study
GP3274	*trpC2 cspC*-A58P-*spec* Δ*cspD::aphA3*	This study
GP3275	*trpC2 cspC*-A58P-*spec* Δ*cspB::tet* Δ*cspD::aphA3*	GP3251→GP3274
GP3283	*trpC2 amyE::*(P*_cspB_*-*lacZ cat*)	pGP3136→168
GP3286	*trpC2 amyE::*(P*_cspD_*-*lacZ*-*cat*)	This study

^1^ The genomic DNA of these strains was analyzed by whole-genome sequencing.

**Table 2:** Promoter activities of the *B. subtilis csp* genes.

Strain	Promoter	Enzyme Activity in Units/Mg of Protein ^a^	
		15 °C	37 °C
GP3283	*cspB*	14,900 ± 1680	11,750 ± 560
GP1984	*cspC*	30,950 ± 2900	6320 ± 280
GP3286	*cspD*	11,300 ± 1600	19,600 ± 450

^a^ All measurements were performed in triplicate. The standard deviations are indicated.

**Table 3:** Effect of the deletion of *cspB* and *cspD* on the expression of *B. subtilis* genes and operons.

Transcription Unit	Function ^1^	Remarks	Fold Regulation Upon *cspB cspD* Deletion
mRNAs with increased amounts upon deletion of *cspB* and *cspD*			
*liaIH*	Resistance against cell wall antibiotics	Activated by LiaR	150
*rbsACB*	Ribose utilization	Repressed by CcpA	110
*maeN*	Malate uptake	Activated by MalR	62
*tlpA*	Chemotaxis receptor	SigD regulon	47
*ywsB*	General stress protein	SigB regulon	45
*manPA-yjdF*	Mannose utilization	Activated by ManR	36
*yodTSR*	Spore metabolism	SigE regulon	32
*yorR*	Unknown, SPβ prophage		30
*ybdN*	Unknown	Repressed by AbrB	23
*uxaC*	Hexuronate utilization	Repressed by ExuR and CcpA	21
mRNAs with decreased amounts upon deletion of cspB and cspD			
*cydABCD*	Respiration, cytochrome bd oxidase	Repressed by CcpA and Rex	−640
*ldh-lctP*	Overflow metabolism	Repressed by Rex	−530
*narGHJI*	Nitrate respiration	Activated by Fnr	−210
*ywcJ*	Putative nitrate channel	Repressed by Rex	−163
*arfM*	Regulation of anaerobic genes	Activated by Fnr	−90
*mhqNOP*	Resistance against oxidative stress	Repressed by MhqR	−64
*cotJC*	Spore coat protein	SigE regulon	−35

^1^ Functional information is based on the *Subti*Wiki database [1].

## Data Availability

RNA-seq data have been deposited in the ArrayExpress database at EMBL-EBI (www.ebi.ac.uk/arrayexpress, accessed on 2 July 2021) under accession number E-MTAB-10658.

## Supplementary Materials

The following are available online at https://www.mdpi.com/article/10.3390/microorganisms9071434/s1, Table S1: Oligonucleotides used in this study. Table S2. Fold changes of the RNA-seq analysis of the cspB cspD double mutant GP1971 as compared to the wild type strain *B. subtilis* 168.
