# Supplementary material for: Functional Redundancy and Specialization of the Conserved Cold Shock Proteins in Bacillus subtilis
Source: Microorganisms. 2021 Jul 2;9(7):1434. doi: 10.3390/microorganisms9071434 (PMC8307031; doi:10.3390/microorganisms9071434)
Supplement: Supplementary file 1 [file microorganisms-09-01434-s001.zip › Table S1.pdf]

Table S1. Oligonucleotides used in this study

Primer	**Sequence 5' → 3'**<br>*Homologous bases for joining PCR are bold*<br>*Restriction sites are underlined*<br>*Shine-Dalgarno sequences are italic*
**Deletion and sequencing of *cspB***	
PF41	ATGATCGGAATTCTGGGGCTG
PF42	**CCTATCACCTCAAATGGTTCGCTG**GCATAAATTGATATGAAAAACTGCAGGTG
PF43	**CCGAGCGCCTACGAGGAATTTGTATCG**TGAAATTTCCTCCTAAAGCGATCATAAC
PF44	ACAGCTTTTAATTTCAGGTGTCTCG
PF45	ACAAAATCATTAGAGGACCGTTTCTTAG
PF46	ATGAGTCCGCCGCTCTTTAG
cat- fwd (kan)	**CAGCGAACCATTTGAGGTGATAGG**CGGCAATAGTTACCCTTATTATCAAG
cat-rev (kan)	**CCGAGCGCCTACGAGGAATTTGTATCG**CCAGCGTGGACCGGCGAGGCTAGTTACCC
Tc fwd2	**CAGCGAACCATTTGAGGTGATAGG**GCTTATCAACGTAGTAAGCGTGG
Tc rev	**CCGAGCGCCTACGAGGAATTTGTATCG**GAACTCTCTCCCAAAGTTGATCCC
**Deletion and sequencing of *cspC***	
PF47	GAAGGGCAGGTACGGAAGATAG
PF48	**CCTATCACCTCAAATGGTTCGCTG**TTGTTGCCTCCTAGTGTGTAACC
PF49	**CCGAGCGCCTACGAGGAATTTGTATCG**TCTTCAATCGTTTATACAAACAGGCTC
PF50	TACGACCAGTTACCGATATACTTGC
PF51	TGAACAGGAGATTTAATGCTTTCTGATG
PF52	ACGGAGCAGGTATAATTGAAGCC
spec-fwd (kan)	**CAGCGAACCATTTGAGGTGATAGG**GACTGGCTCGCTAATAACGTAACGTGACTGGCAAGAG
spec-rev (kan)	**CGATACAAATTCCTCGTAGGCGCTCGG**CGTAGCGAGGGCAAGGGTTTATTGTTTTCTAAAATCTG

**Deletion and sequencing of *cspD***	
MB17	CGCCGAACTGGAAGAGTCATTCC
MB18	**CCTATCACCTCAAATGGTTCGCTG**GTTGAACCATTTTACTTTACCGTTTTGCAT
MB19	**CCGAGCGCCTACGAGGAATTTGTATCG**GTAATCGTGGACCTCAAGCTTCTAATGTTG
MB20	GAAGCACTCCTTGAATCGCTGAAGC
MB21	GGCGAACTTGTCGATGAACATCAG
MB22	GGCAGCTGGCCTTGTTATGATC
kan fwd	AAAGAATTCGATAAACCCAGCGAACCATTTGA
kan rev	TTTGAATTCATCGATACAAATTCCTCGTAGGC
**Deletion and sequencing of *veg***	
PF116	TCAAGAGTCAATATTCATGCGCTTG
PF119	CTGGTGGCAGTGAAAAAGGATG
PF120	**CCGAGCGCCTACGAGGAATTTGTATCG**TTGCATCCACCTCACTACATTTATTG
PF121	**CCTATCACCTCAAATGGTTCGCTG**TTGTTTACTGCTTTTTGTTTTGCCC
PF122	GAAACGTCAGAGCCAATTTCCG
PF123	GTTTCGAATTATAGGAATAGAGCAAACAAG
PF124	AGCAGTTGAAACACCGATTGTC
mls-fwd	**CAGCGAACCATTTGAGGTGATAGG**GATCCTTTAACTCTGGCAACCCTC
mls-rev	**CGATACAAATTCCTCGTAGGCGCTCGG**GCCGACTGCGCAAAAGACATAATCG
**Construction of P_{cspB}-*lacZ* fusions**	
PF151	TTTGAATTCTCATACGCTCTCTTAGTTGATAAACGT
PF152	TTTGGATCCAACATGAAATTTCCTCCTAAAGCGATC
**Construction of P_{cspC}-*lacZ* fusion**	
PF97	TTTGAATTCAGGGGGCTTTGCGATTGAG
PF98	TTTGGATCCTCTAAAGATTTGAATCCGTCACTTTGG

**Construction of P$_{cspD}$-*lacZ* fusion**	
PF246	AGGGTATGTTTCTCTTTGATGTCTTTTTG
PF247	CGGCAATAGTTACCCTTATTATCAAGATAAG
PF248	**CTTATCTTGATAATAAGGGTAACTATTGCCG**TCAGCCATCAATAAAAGCGGTTAC
PF249	**GTCACGACGTTGTAAAACGACGGGATCCCC**GTTGAACCATTTTACTTTACCGTTTTGC
PF250	GGGGATCCCGTCGTTTTACAA
PF251	AACAAAATTCTCCAGTCTTCACATCG
**Construction of P$_{veg}$-*lacZ* fusions**	
PF127	AAAGAATTCAGAGAAAGGGCTTGGAGGTATTG
PF118	TTTGGATCCCCTAAATTCCCATCAAGCGATCTTTT
**Construction of CspC[A58P] variant**	
PF178	[5'Phosphorylated]ACGTTAGCAGCTTGAGGTCCACGAG
**Qualitative PCR for *manR-manP, liaH-liaG, pyrR-pyrP, pyrP-pyrB* readthrough transcripts**	
PF190	GTTTAAACAAGCCATTTTGAATCGTGA
PF191	CAATCGCATGAACAGTACCGC
PF192	GAGGTTCGTACTGCCAGATCAC
PF193	GCTGAAGTGGCTGGCAAAC
PF196	GAACGCATTGAACAGATTGAGGG
PF197	CAGCCATCCTGTTCCAAGCT
PF198	CTGAGCAAGGTTGTCGGACA
PF199	AGACGTCCACACCGATTGATTC
**Quantative PCR for *manR-manP, liaH-liaG, pyrR-pyrP, pyrP-pyrB* readthrough transcripts**	
PF201	AGCTGACGAAAGAAACCAATGT
PF202	CGAAATCAGCTCATTAAAATCGC
PF203	ACCGGCAGTGATCAACAGTT
PF204	GCGGCAAATGAATAAGCGGA

PF205	GGTCTTTGTATGCCTCTTTGCG
PF206	CCCAAGAGAAAGGTGTCGGG
PF207	CAGAGAGGCTTGGAAGGGTT
PF208	GCTAAGTTCACTCATCGTCGT
